# Two Forms of Functional Reductionism in Physics

**DOI:** 10.1007/s11229-024-04507-0

**Published:** 2024-02-15

**Authors:** Lorenzo Lorenzetti

**Affiliations:** Department of Philosophy, University of Bristol, Cotham House, Bristol, BS6 6JL UK

**Keywords:** Reductionism, Intertheoretic reduction, Intertheory relations, Functionalism, Functional reduction, Thermodynamics, Statistical mechanics

## Abstract

Functional reductionism characterises inter-theoretic reduction as the recovery of the upper-level behaviour described by the reduced theory in terms of the lower-level reducing theory. For instance, finding a statistical mechanical realiser that plays the functional role of thermodynamic entropy allows to establish a reductive link between thermodynamics and statistical mechanics. This view constitutes a unique approach to reduction that enjoys a number of positive features, but has received limited attention in the philosophy of science. This paper aims to clarify the meaning of functional reductionism in science, with a focus on physics, to define both its place with respect to other approaches to reduction and its connection to ontology. To do so, we develop and explore two alternative versions of functional reductionism, called *Syntactic Functional Reductionism* and *Semantic Functional Reductionism*, that expand and improve the basic functional reductionist approach along different lines, and make clear how the approach works in practice. The former elaborates on David Lewis’ account, is connected with the syntactic view of theories, employs a logical characterisation of functional roles, and is embedded within Nagelian reductionism. The latter adopts a semantic approach to theories, spells out functional roles mainly in terms of mathematical roles within the models, and is expressed in terms of the related structuralist approach to reduction. The development of these frameworks has the final goal of advancing functional reductionism, making it a fully developed account of reduction in science.

## Introduction

Functionalism is all about understanding things in terms of the roles they play. According to this view, theoretical terms are defined by the roles they have in theories, and properties are cashed out in terms of their causal roles or behaviour. Functional reductionism exploits functionalism to shed light on inter-level relations: finding a lower-level realiser for an upper-level functional role gives us a way to connect the two levels.^1^

Functional reductionism is a view with a venerable tradition in the philosophy of mind (e.g. Kim, 1998, 2005; Lewis, 1972; Morris, 2020), where it has been employed to relate phenomenal and mental states. If pain is that state “that tends to be caused by bodily injury, to produce the belief that something is wrong with the body [...]” (Levin, 2021) and so on, and we individuate a brain state that fills those roles, we can functionally reduce pain to that specific kind of physical state.

This account is growing in importance within the philosophy of science as well, especially in the philosophy of physics (e.g. Butterfield & Gomes, 2022, 2023; Esfeld & Sachse, 2007; Huggett and Wüthrich, 2021; Lam & Wüthrich, 2018, 2020; Lorenzetti, 2022, 2023; Robertson, 2022).^2^ In this context, functional reductionism is primarily used to model cases of theoretical reduction between scientific theories and represents a unique approach to reduction.^3^ It has been used for instance to model reductive relationships between thermodynamics and statistical mechanics, between classical and quantum mechanics, and between general relativity and quantum gravity theories. According to functional reductionism, the primary aim of reduction is to find the right lower-level realisers for the upper-level behaviour: reduction is secured if we find in the bottom-level theory some theoretical elements that play the functional roles described by the upper-level theory. For instance, let’s say we can functionally define ‘temperature’ in terms of its role within thermodynamics, and we find out that ‘mean kinetic energy’ plays the role of temperature: in that case, we can functionally reduce temperature to mean kinetic energy, and this can be regarded as a step in the reduction of thermodynamics to statistical mechanics.

The aim of this paper is to advance the literature on functional reductionism in science by making clear how this approach to reduction works, elaborating on the way in which reduction is exactly achieved according to the view, on its relationship with other standard approaches to scientific reduction, and on its connection with ontology. We focus in this essay on functional reductionism in physics, and we take as our starting point and case study an instance of functional reduction recently advanced by Robertson (2022), concerning the reduction of thermodynamic entropy to statistical mechanics.

In order to develop functional reductionism, we first elaborate on and review in detail the most fully developed functional reductionist account in the literature, introduced by Lewis (1970) and recently defended by Butterfield and Gomes (2023). We call this approach *Syntactic Functional Reductionism*. It is a version of functional reductionism embedded within a syntactic view of theories, especially as it employs a logical characterisation of functional roles via Ramsey sentences, and is a form of Nagelian reduction. We apply the view to our case study and present some possible shortcomings of this approach. We thus introduce a novel alternative framework for functional reductionism, called *Semantic Functional Reductionism*. It draws on certain features of the semantic view of theories, spelling out functional roles in terms of the (mainly mathematical) models of the theory, and is formulated in terms of a structuralist approach to reduction. We show how this framework accounts for our case study, and find that it can overcome the issues faced by the version of Syntactic Functional Reductionism we have presented.

The syntactic and semantic qualifications are adopted here in a specific sense. Syntactic Functional Reductionism qualifies as syntactic in a narrow sense of the term, mostly in virtue of its use of predicate logic in formulating functional roles. At the same time, we call the other view semantic in virtue e.g. of the centrality of mathematical models in the formulation of functional reductionism, but this does not rule out the possibility of syntactic-friendly views including this feature. The Semantic label is instead a way to stress the contrast of that approach with an account that is as traditionally syntactic as the Lewisian one, and we grant that alternative versions of Syntactic Functional Reductionism differing from the Lewisian specific approach are in principle viable.

The primary results of this paper are thus to pose some potential issues for the standard approach to functional reductionism and to put forward a new way to explicate functional reductionism. Overall, both views remain viable in principle, as they each enjoy particular strengths and weaknesses that the following discussion will bring about, and thus the final goal of the paper is to develop two alternatives that can serve as starting points for future works. In this sense, the two accounts we are presenting are meant to stand at two opposite ends of a spectrum, leaving open the possibility of implementing features of each approach into the other.

The project of this paper, as just described, contributes to the literature on reduction in several important ways. **First**, this essay takes a crucial step toward the establishment of functional reductionism as a fully developed alternative account of inter-theoretic reduction in science, and will therefore have an impact on both the specific literature on functional reductionism and the general literature on reductionism in science. In fact, it clarifies the debate on functional reductionism by providing and analyzing two clear alternative frameworks according to which we can articulate the view. As mentioned, the discussion of the frameworks leads us to show how we can develop in more detail the notion of functional role in different ways, sheds light on the relationship between ontology and reduction within functional reductionism, and makes clear the connection between functional reductionism and the other standard approaches to reduction.

**Second**, relatedly, by discussing the thermodynamics case study, we show how functional reduction works in practice, and we further our understanding of the view by analyzing both the advantages and disadvantages of each framework and the ontological bearings of each specific approach. Syntactic Functional Reductionism delivers an approach to ontology that is very clear but also very rigid, whereas the Semantic alternative allows for a more flexible view of the ontological aspects of reduction. These aspects mirror the situation at the theoretical level: while the former approach adopts a very rigorous and logically-formulated view on the formulation of theories, the latter embraces a model-based account. The choice between the two frameworks hinges also on these features. Most importantly, the lack of flexibility characterising the Syntactic Functional Reductionist approach is problematic insofar as we want to freely choose which elements of the theory we want to be realists about, and the logical translation aspect of the view can work against the framework as well, whereas the Semantic Functional Reductionism works better in these respects. The present paper thus not only develops two alternative takes on functional reductionism, but also assesses them via the analysis of a realistic case study and allows us to provide a thorough evaluation of each alternative.

**Third**, proposing an alternative to the extant Lewisian syntactic-based account of functional reductionism is an important improvement for the whole functional reductionist approach. Indeed, someone could find endorsing functional reductionism problematic just due to contingent issues related to the specific Lewisian account, as that is currently the only complete framework for the view. Providing an alternative, represented here by Semantic Functional Reductionism, makes functional reductionism much more resistant to this kind of risk and makes functional reductionism more palatable overall.

**Fourth**, more generally, the essay is intended to have a broader impact on the whole debate on theoretic reduction, as we show that functional reduction can integrate either Nagelian or structuralist reduction and provide a revised and improved version of these approaches, embedded in the functional reductionist framework. Syntactic Functional Reductionism is indeed a form of Nagelian reduction in which bridge laws are not postulated as additional assumptions, and are thus less problematic, while Semantic Functional Reductionism improves the structuralist account of reduction, providing a more precise version of it and a stronger justification for the approach. Thus we don’t simply clarify the place of functional reductionism with respect to other accounts of reduction, but we also argue that the two forms of functional reductionism presented here can be considered to be improved versions of, respectively, Nagelian and structuralist reduction.

Section 2 reviews Robertson’s functional reductionist proposal concerning thermodynamics, which will be the starting point of our discussion. Having presented how functional reduction works for a real example, in Sects. 3 and 4 we discuss the two functional reductionist frameworks. Section 5 overviews the overall pros and cons of each approach and explores how the two accounts could be further developed and combined.

## A case study for functional reductionism

We review here the instance of functional reduction recently put forward by Robertson (2022). Being a recent and very precise example of scientific functional reduction in a highly debated area, it provides a good introduction to the approach, and a well-suited case study for our discussion about the relationship between functional reductionism and ontology in Sects. 3.3 and 4.2.

Robertson’s aim is to reduce the thermodynamic entropy $S_{TD}$ to some statistical mechanic quantity, as a step in the reduction of thermodynamics to statistical mechanics—in particular, to reduce the second law of thermodynamics, which can be expressed in terms of the behaviour of the thermodynamic entropy.^4^ To do so, her goal is to find in statistical mechanics a realiser for the role of the thermodynamic entropy. We report here just the essential details. Let’s start from the top-level theory, and in particular from thermodynamic entropy $S_{TD}$. This is a function of the state of a thermodynamic system, like pressure, and it is roughly said to measure the ‘disorder’ of the system.^5^ Or, using Clausius’ definition, entropy can be defined as the thing that increases by Q/T whenever heat Q enters a system at temperature T.^6^ We can thus represent the change of entropy $dS_{TD}$ in a system as:

1 $$\begin{aligned} \dfrac{dQ}{T}=dS_{TD}, \end{aligned}$$

where *dQ* is the change in heat (the heat absorbed) and T is the temperature. Thermodynamic entropy can then be represented by integrating (1). In this way we represent the entropy difference between two states of the system, in this case state 0 and state B:

2 $$\begin{aligned} \int _{0}^{B} \dfrac{dQ}{T}=S_{TD}(B). \end{aligned}$$

This quantity is crucial for modelling thermodynamic behaviour, and thus reducing it to statistical mechanics would be an essential step to reducing thermodynamics to statistical mechanics, as we can use this to formulate the second law of thermodynamics. The two central characteristic features of $S_{TD}$ on which reduction is focused are related to how this function works in two kinds of situations.

On the one hand, let’s look at the case of arbitrary quasi-static reversible cycles in the equilibrium space $\Xi$. A thermodynamic equilibrium state is a state in which no macroscopic change occurs in a system, and the equilibrium space is the space of those states. A quasi-static reversible cycle is a process in which the system moves through equilibrium states, thanks to the fact that it is evolving slowly. For these processes we expect the following to occur:

3 $$\begin{aligned} \oint \dfrac{dQ}{T}=0. \end{aligned}$$

That is, if a process P is a quasi-static reversible process, we can write:

4 $$\begin{aligned} \Delta S_{TD}=0. \end{aligned}$$

On the other hand, it can be proven that, if a process P (say, between state A and state B) is not quasi-static, the thermodynamic entropy is a quantity that cannot decrease:

5 $$\begin{aligned} S_{TD}(B)-S_{TD}(A) \ge 0, \end{aligned}$$

that is:

6 $$\begin{aligned} \Delta S_{TD} \ge 0. \end{aligned}$$

Functional reduction consists in finding a statistical mechanics realiser—a statistical mechanics function or quantity—for the roles of $S_{TD}$ which are mathematically specified by (4) and (6). Using words to express that theoretical functional role, we can say that we have to “Find a statistical mechanics realiser which, for thermally isolated systems, is increasing in non-quasi-static processes, but non-increasing in quasi-static processes, such as those represented by curves in $\Xi$” (Robertson, 2022, p. 21).

Let’s thus move to statistical mechanics.^7^ A key concept in statistical mechanics is that of canonical ensemble, which is used to represent the possible states in which a system can be. In particular, the canonical ensemble gives the probability that a system is in a specific state *n*:

7 $$\begin{aligned} p(n)=\dfrac{e^{-E_{n}/k_{B}T}}{\sum _{m}e^{-E_{m}/k_{B}T}}, \end{aligned}$$

where E is the energy of each state and $k_{B}$ is Boltzmann’s constant. To simplify, we can introduce a new notation, and write $\beta \equiv 1/k_{B}T$ and $Z=\sum _{n} e^{-\beta E_{n}}$. We can thus rewrite (7) as:

8 $$\begin{aligned} p(n)=\dfrac{e^{-\beta E_{n}}}{Z}. \end{aligned}$$

Moving to quantum statistical mechanics, we write (8) in a slightly different way. In quantum mechanics, a system can be described via a density matrix $\rho$. We can thus express the canonical ensemble for a given system as:

9 $$\begin{aligned} \rho =\dfrac{e^{-\beta \hat{H}}}{Z_{q}}, \end{aligned}$$

where $\hat{H}$ is the Hamiltonian operator representing the energy and the new $Z_{q}$ is the quantum version of the partition function.^8^ In both classical and quantum statistical mechanics, the canonical ensemble can be used to represent thermal equilibrium. What matters for us is that $\rho$ is important to the introduction of a new quantity, the quantum Gibbs entropy, since the canonical ensemble is said to maximise Gibbs entropy $S_{G}$:

10 $$\begin{aligned} S_{G}=-k_{B}Tr \rho \text {ln} \rho , \end{aligned}$$

where *Tr* is the trace over the density matrix. Having introduced $S_{G}$, we shall now gloss over a lot of details and just report here how the reduction of thermodynamic entropy is achieved through a functional reduction of $S_{TD}$ to $S_{G}$. Briefly put, Robertson (2022, §6) shows that, for quasi-static processes in quantum statistical mechanics, we can write:

11 $$\begin{aligned} \Delta S_{G}=0 \end{aligned}$$

On the other hand, for non-quasi-static adiabatic processes, with $t>>t_{1}$, we can derive^9^:

12 $$\begin{aligned} S_{G}[\rho _{can}(t_{1})]-S_{G}[\rho _{can}(t_{0})]>0. \end{aligned}$$

The presentation so far provides what we asked for: we have found a statistical mechanical function—i.e. the statistical mechanical entropy $S_{G}$—that is constant in quasi-static processes (11) and that increases in rapid non-quasi-static processes (12). Indeed, the statistical mechanical equations (11) and (12) for $S_{G}$ mathematically mirror the thermodynamic equations (4) and (6) embedding $S_{TD}$. The result is that these equations display the functional similarities shared by the two quantities.

Finally, to strengthen the functional correlation between the two quantities, Robertson shows that, in the right parameter regime, $S_{TD}$ and $S_{G}$ evolve in a very similar way. First, take (1), and derive the following from the first law of thermodynamics $dE=TdS-pdV$, where *V* is the volume:

13 $$\begin{aligned} dS_{TD}=\dfrac{1}{T_{TD}}(dE_{TD}+p_{TD}dV). \end{aligned}$$

On the other hand, within Gibbsian quantum statistical mechanics, given certain assumptions and approximations, we can derive Gibbs entropy as:

14 $$\begin{aligned} dS_{G}=\dfrac{1}{T}(d\langle E \rangle +\langle p \rangle dV), \end{aligned}$$

where the brackets denote that we are taking the average value. All in all, we can conclude that the Gibbs entropy functionally reduce the thermodynamical entropy:

The Gibbs entropy can play the right role, since it increases in non-quasi-static processes but is constant in quasi-static processes. Furthermore, $S_{G}$ is connected to heat in the right way (Robertson, 2022, p. 31).

To recap, Robertson’s goal was to find a statistical mechanical reductive basis that could reduce a specific thermodynamic behaviour, which is codified by the evolution of the thermodynamic entropy $S_{TD}$. To do so, she exploited the functionalist idea that, in order to reduce the thermodynamic entropy to statistical mechanics we have to find a statistical mechanical quantity which—at least approximately—plays the role of $S_{TD}$ in the upper theory. The functionalist model of reduction thus tells us here what we have to do if we want to establish reduction, that is we have to focus on finding something in the low-level theory that instantiates the right patterns of behaviour within the high-level theory. In this way, functional reductionism provides a clear and plausible model for reduction, that we can use to find a statistical mechanical underpinning for thermodynamics. Indeed, as stressed by Robertson, formulating a reductionist account for the second law of thermodynamics is a notoriously difficult task,^10^ and functional reductionism provides the tools to do so.^11^

Sections 3 and 4 develop two alternative frameworks for functional reductionism, and this case study will be illustrative to discuss how each view works. We see that Syntactic Functional Reductionism faces the problem of shoehorning the mathematical formalism used here into a logical formulation and the problem of accounting for the approximation required for the reduction. On top of that, the way in which the framework reformulates this example of functional reduction prompts a very specific, but also too restrictive, account of the ontological implications of the reduction. On the contrary, the formulation of functional reductionism provided by the Semantic framework is model-based and mostly mathematically formulated, and thus accommodates in a more straightforward way the case study as presented here. Framed in that way, the account also allows for a more flexible account of the ontological meaning of the reduction at stake.

## Syntactic functional reductionism

The first functional reductionist approach we introduce is called ‘Syntactic Functional Reductionism’. It is based on the functional reductionist account first put forward by Lewis (1970) and recently defended by Butterfield and Gomes (2023), and is currently the most developed functional reductionist account available in the philosophy of science literature.^12^ According to this approach, reduction goes as follows. The first step is to write down the laws of the reduced theory in terms of the reducing theory. At that point, by appealing to functionalism, we derive the bridge laws between the theoretical terms of the two theories from the laws of the bottom theory alone. We are thus able to derive the upper-level laws from the bottom-level laws plus bridge laws. Since law-derivation via bridge laws is the essence of Nagelian reduction, this approach can be considered a kind of Nagelian reduction. However, within this functionalist form of Nagelian reduction, bridge laws are derived from the reducing theory, and not added as extra postulates, like it is in the standard Nagelian view. Because of this feature, the account can be regarded as an improved version of Nagelian reduction.^13^

The aim of this section is to present the most developed version of the account possible and then provide an assessment of the framework. Section 3.1 introduces the basis of Syntactic Functional Reductionism, i.e. David Lewis’s account, shows its connection with Nagelian reduction and with the syntactic view, and describes the link between theoretical reduction and ontological reduction within the account. Section 3.2 delves further into the account, showing how the basis can be improved with respect to two aspects: dealing with approximation and moving to a more local kind of reduction. Section 3.3 applies the case study of Sect. 2 to Syntactic Functional Reductionism as developed in Sects. 3.1 and 3.2 and raises some issues for the view.

### The core: the Lewisian basis

This subsection introduces in more detail the core of Syntactic Functional Reductionism, as defended by Lewis, Butterfield, and Gomes. Since the view is a kind of Nagelian reduction, it is important to first briefly introduce the latter account. According to Nagel’s (1962) classic model of reduction, a theory $T_{P}$ can be said to be reduced to another theory $T_{F}$ iff the laws of $T_{P}$ can be deduced from the laws of $T_{F}$ plus some auxiliary assumptions. In the (common) case in which the two theories do not share their theoretical terms we need also to postulate bridge laws connecting the two vocabularies. For instance, in the context of the reduction of thermodynamics to statistical mechanics, we can derive the Boyle–Charles law from statistical mechanics’ laws given a bridge law stating that ‘temperature’ means ‘mean kinetic energy’ (cf. Dizadji-Bahmani, 2021).

The Lewisian approach provides a special Nagelian account of reduction that builds on functional reductionism in order to obtain the required bridge laws. According to this view, inter-theoretical reduction essentially proceeds in three steps:

1. We write down the laws of theory T in logical terms, then we replace all the theoretical terms $$\tau _{1}...\tau _{n}$$ of the theory with open variables $$x_{1}...x_{n}$$, leaving just non-theoretical terms and connectives, i.e. we move from $$ T (\tau _{1},...\tau _{n})$$ to *T*($$x_{1},...x_{n})$$. We now build the Ramsey sentence of the theory by placing an existential quantifier in front of the sentence: $$ \exists x_{1},...x_{n} T (x_{1},...x_{n})$$. This says that there are certain *x*s which realise the theory. On the assumption that the theory is uniquely realised (i.e. there is only one set of $$x_{1}...x_{n}$$ that realises the theory),^14^ we can construct explicit functional definitions of the $$\tau _{1}...\tau _{n}$$ via the Ramsey sentence. These says e.g. that $$\tau _{i}$$ is ‘that thing that occupies the $$x_{i}$$-role within the theory’.
2. We find another theory T* embedding new theoretical terms $\rho _{1}...\rho _{n}$. Suppose that the following sentence is a theorem of T*: $T[\rho _{1}...\rho _{n}]$.^15^$T[\rho _{1}...\rho _{n}]$ does not contain $\tau$-terms, and it says that the original theory *T* is realized by a *n*-tuple $\rho _{1}...\rho _{n}$, taken from T*. In case T is uniquely realised by the *n*-tuple $\rho _{1}...\rho _{n}$, Lewis shows that we can functionally define the $\rho _{i}$ as the occupiers of certain *x*-roles in T, and those functional definitions are theorems of T*.^16^
3. Following step (2), we can derive theoretical identifications $\rho _{1}=\tau _{1}, \ldots , \rho _{n}=\tau _{n}$ by transitivity of identity. These are *bridge laws* and they play the role of Nagelian bridge laws in the theory derivation of T from T*.

More informally, the process goes as follows: we first specify the theoretical roles of the theoretical terms within a theory T via the Ramsey sentence of a theory, i.e. we build functional definitions for the terms $\tau _{1}...\tau _{n}$ in the theory. Then, we find a second theory T*. This theory can realise the former theory T in terms of $\rho _{1}...\rho _{n}$, and so we show that it contains theoretical terms $\rho _{1}...\rho _{n}$ which play the roles of the entities $\tau _{1}...\tau _{n}$. Thus, on the assumption that the Ramsey sentence of theory T is uniquely realised, we deduce bridge laws between the two theories, i.e. we connect the vocabularies of the two theories. This happens because we have terms that fall under the same functional profile and thus they can be identified thanks to functionalism. If a term $\tau _{i}$ is identified to a term $\rho _{i}$ in this way, we say that $\tau _{i}$ is functionally reduced to $\rho _{i}$. The functional reduction of the theoretical terms within different theories is thus a step in the full derivation of the laws of the reduced theory from the reducing theory’s laws.

Hence Lewisian reduction is a special form of Nagelian reduction in which theory deduction is couched in terms of logical derivation and in which bridge laws are functionally derived and thus deduced, as opposed to postulated as additional empirical hypotheses.

We can also see now how this functional reductionist view fits naturally within a traditional understanding of the syntactic view of theories. One key feature usually attributed to the syntactic approach is the claim that “the structure of a scientific theory is its reconstruction in terms of sentences cast in a meta-mathematical language” (Winther, 2021).^17^ Since a prerequisite of the Lewisian account is the idea of expressing scientific theories as sentences formulated in the language of first-order logic, the account arguably falls within a traditional understanding of the syntactic side. Moreover, within this approach to theories, inter-theoretical relations such as reductive relations between theories are normally formulated as deductions under a given class of logical relations, and the account of reduction presented above presents this feature. We can thus appreciate how the topic of the nature of scientific theories heavily influences functional reductionism, since the stance we take on theories is crucially correlated to the way in which we cash out functional roles, which are here defined via the Ramsey sentence using predicate logic. We shall see that the same is true for Semantic Functional Reductionism, which falls within the semantic approach in many respects. It should be stressed that we are not attempting nor requiring here a complete and accurate reconstruction of the debate on the nature of theories, especially because many versions of each view are available, and the distinction between the two is often blurry. Rather, we just aim to present them in a way that can help us highlight the difference between the two brands of functional reductionism discussed in the paper.

Moving on, let’s discuss the relationship between this account of inter-theoretic reduction and the ontology of the theories it concerns. Notice that here and in the rest of the paper we will be careful in distinguishing between formal mode and material mode, i.e. in discussing reductionism at the level of theories and at the level of ontology.^18^ Functional reductionism, as discussed so far in this section, is clearly a form of reductionism about theories. However, in Lewis’s account, this functional reductionism about theories, which leads to identity relations between theoretical terms, is meant to be a way to ensure functional reduction about ontology as well. Lewis makes this clear in several places, for instance:

The *T*-terms have been defined as the occupants of the causal roles specified by the theory *T*; as *the* entities, whatever those may be, that bear certain causal relations to one another and to the referents of the *O*-terms (Lewis, 1972, p. 255).

The passage from the formal mode to the material mode is thus straightforward here. On the assumption that the theoretical terms refer to actual entities, the theoretical functionalisation is just a means to codify in a scientifically accurate way the roles played by the worldly entities referred to by the theoretical terms. That is, functional reduction of theoretical terms can be a guide to functional reduction of entities. Lewis is explicit about this. For him, theoretical terms like ‘electron’ are meant to refer to an actual entity, as he wants to maintain a clear form of scientific realism.^19^ Thus, when we functionally define a theoretical term in the upper theory and we find some other theoretical term in the bottom theory with the same role, we should believe also that there is a bottom entity (referred to by the term $\rho _{i}$) to which the upper entity denoted by $\tau _{i}$ is reduced to.

It should be noticed that this is a form of realiser functionalism, since the functionalised entity at the top is type-identified with its realiser at the bottom. This is actually a radical consequence of the account which should be stressed: on the ontological level, the Lewisian account leads us to maintain identity relations between the reduced and the reducing entity. When the entity belonging to the bottom level behaves in the right way, *that same entity* turns out to be the upper-level entity which is the target of the reduction, in virtue of the fact that it plays the role of the target entity.^20^

### Two improvements of the Lewisian basis

The Lewisian account presented above is a framework that makes the broad functional reductionist approach more precise. It presents a formal way to spell out the notion of functional role at the theoretical level (via Ramsey sentence), embeds a specific approach to reduction (Nagelian reduction), and shows a close connection with a specific view of scientific theories (the syntactic one). This subsection presents two improvements on the Lewisian core. This improved version can be taken as the real basis of Syntactic Functional Reductionism. We discuss (1) the move from a Nagelian to a Neo-Nagelian model, and (2) the move to a more local version of functional reductionism.

The first aspect concerns the role of approximations in reduction. We start by pointing out that the commonly adopted version of Nagelian reduction is not the classic model proposed by Nagel (1962) and introduced in the last subsection, but a more refined approach that has been put forward by Schaffner (1967) and recently by Dizadji-Bahmani et al. (2010).^21^ This ‘Neo-Nagelian’ account relaxes the derivability criterion and argues that, to ensure reduction, it is sufficient to derive laws that are *approximately* the same as the laws of the original theory $T_{P}$. More precisely, according to this view, $T_{F}$ reduces $T_{P}$ iff we can build a theory $T_{P}^{*}$—which is a corrected version $T_{P}$ standing in a relation of ‘strong analogy’ with $T_{P}$—which is derivable from $T_{F}$ given some appropriate auxiliary assumptions and bridge laws. The reason is that it is almost never the case that we can derive the exact laws of an upper theory (to be reduced) from a bottom theory. At most, we can recover the behaviour described from the top theory in an approximate way and just in particular situations. Why is it important to point this out here? The reason is that the issue behind the introduction of the Neo-Nagelian approach affects the Lewisian account of functional reduction as well, qua Nagelian-based account, even though this approach does not require postulated bridge laws. In fact, the Lewisian process of reduction requires the deduction of the reduced theory’s laws from the reducing theory’s laws, and requires us to express the terms $\rho _{i}$ as playing the role of the $\tau _{i}$. However, if it is true that we cannot ever exactly deduce the original reduced theory, but just an approximate version of it, then also the Lewisian view needs to be amended like the classic Nagelian approach. We should thus expect to replace the reduced theory with a strongly analogous version of it, and accordingly, we should expect the $\rho _{i}$ to functionally realise some terms that are not strictly speaking our original $\tau _{i}$ but rather terms that behave approximately like them.

For instance, if we are dealing with the reduction of classical mechanics to quantum mechanics, we cannot expect quantum systems to behave exactly as classical systems, but only approximately so. Therefore, if we want to functionally define e.g. two-particle classical systems in terms of their role in Newtonian mechanics to reduce them, we will need to describe their behaviour through a modified version of Newton’s law taking into account fluctuations in the dynamical evolution of the system, instead of Newton’s law. Only then, once we have defined two-particle classical systems in terms of their approximate role in classical mechanics, we can really find an appropriate realiser within the quantum domain and satisfy the uniqueness requirement set by the account.

We should note that the need to take approximations into account is already acknowledged by Lewis (1970, pp. 445–446) himself in the development of the account, but we have presented the issue here since (i) the importance of approximation goes often unmentioned in the more recent literature on functional reductionism^22^ and (ii) whereas Lewis simply talks about ‘near-realisation’ of the reduced theory’s terms, the present treatment has the merit of implementing approximations in Lewisian reduction within the wider context of Neo-Nagelian reduction as developed by Schaffner and others.

Moving to our second topic, we can draw an important distinction within the Syntactic Functional Reductionist framework between a global and a more local version of the view. In the context of inter-theoretic reduction, a global approach is concerned with the reduction of the whole top-level theory to the bottom-level theory, whereas a local approach to reduction focuses primarily on the reduction of specific models or specific parts of the theory. As a version of Nagelian reduction, which in principle concerns whole-theories reduction via the deduction of the top-level laws from the bottom-level laws, the Lewisian approach presented so far technically qualifies as a form of global reduction. However, we highlight that the view can easily be turned into a more local approach. In fact, once we have logically expressed the theory and derived the Ramsey sentence, we are actually free to functionalise either every theoretical term in the theory or just some of them. In this second case, we can provide functional definitions just for one or some ‘problematic’ terms, and perform a functional reduction only for them. The passage from formal to material mode then goes as usual: once a specific term is functionally reduced, we can take the formal functional reduction as representing an ontological functional reduction.

A reason to prefer the local approach comes from its practical viability and flexibility, as we are not expected to functionally reduce every term in the top-level theory at stake, but rather we can focus on the reduction of specific phenomena described by the theory. Most importantly, this characterisation of Syntactic Functional Reduction reflects more accurately how functional reduction is employed in practice in the modern literature in physics. For instance, this is the approach adopted by Butterfield and Gomes (2023) and Lorenzetti (2022) when they apply Lewisian functional reduction to their case studies, and is more representative of the functional reductionist approach of Robertson (2022) as presented in Sect. 2.^23^ Furthermore, framing this functional reductionist kind of Nagelian reduction in a more local way makes the view consistent with the less restrictive reading of Nagelian reduction that has been recently provided in the literature, e.g. by van Riel (2011), who stresses that “according to Nagel, reduction of models, fragments of theories, isolated statements, and so forth, is not excluded in principle” (van Riel, 2011, p. 361).

### Thermodynamics and syntactic functional reductionism

Section 2 presented the reduction of the second law of thermodynamics via the functional reduction of thermodynamical entropy to Gibbs entropy. This is an example of a local functional reduction of the upper-level quantity $S_{TD}$ to the lower-level quantity $S_{G}$. We employ now this case study to analyse Syntactic Functional Reductionism. We discuss the example both at the formal and the material levels, and raise three possible shortcomings of Syntactic Functional Reductionism: a *translation* issue, a challenge related to *approximation*, and finally an *ontological* problem. These challenges are meant to be general issues for the version of Syntactic Functional Reductionism presented here and we are employing this case study mainly to show how they arise in more detail.

To begin with, let’s look more closely at how the Lewisian approach can be applied to the case study at the level of theoretical reduction, at least in principle. The first step is the translation of the laws of thermodynamics from mathematics to predicate logic. Assuming this is possible, we then focus on the theoretical term $\tau$ denoting ‘thermodynamic entropy’ which is represented by $S_{TD}$ in the equations. The idea is to use the Ramsey sentence to build an explicit definition for that term as ‘that thing that occupies the *x*-role within the theory’, where the role is determined by the nomological relations of the term expressed in the Ramsey sentence and taken from Eqs. (4), (6), (13). To be more precise, the Ramsey sentence cannot be constructed from (13), because we know that (14) is only approximately structurally similar to (13) and thus we need to first obtain an approximate version of (13). The second step is to repeat the process for statistical mechanics and the term $\rho$ denoting ‘Gibbs entropy’, building a definition for the term via the logically reformulated equations (11), (12), (14). Showing that we can match the definitions, the two theoretical terms are identified and thus bridge laws are obtained.

We thus move to a critical analysis of the approach. In principle, this is a consistent project, but we immediately face two challenges. First, the whole translation process is not merely a challenging and complex task, but it could be taken to be complex in a futile or avoidable way. This aspect of the framework comes from the specific syntactic approach underlying the Lewisian basis of the account, but the translation passage could be seen more as an unnecessary attempt to shoehorn the mathematical formalism into the language of first-order logic, than as a genuinely useful step within the reduction process. Thus, an alternative functionalisation strategy that does not presuppose this passage would be preferable, other things being equal. Second, we have seen in the previous section that the higher-level theory we are meant to logically translate is not really thermodynamics, but rather an approximate version of thermodynamics, or another theory standing in a relation of strong analogy with it. Building such a theory is not a trivial task, and thus this adds an additional burden to the process of functional reduction, above the logical translation. In particular, since we are here dealing with theories as logically formulated sets of sentences, we cannot simply directly appeal to mathematical notions of approximation between models, but rather we have to rely on a syntactic-based form of approximation.^24^ Section 4 shows that Semantic Functional Reductionism fares better than the Syntactic framework in both this latter respect and the previous one in providing a more minimal account, *ceteris paribus*.

Moving to the connection between theoretical functional reductionism and ontology, a puzzle can be presented with respect to our case study, if one adopts a scientific realist attitude (as Lewis does). In fact, whereas $S_{TD}$ can be interpreted as a property of an individual system, $S_{G}$ is defined as a property of a probability distribution over possible micro-states, i.e. a property of an ensemble. In this sense, it is not clear if the step from formal to material is warranted. Even if we grant the success of functional reduction at the theoretical level, it is prima facie difficult to see how to translate the functional reduction from theoretical quantities to physical properties, since we are supposed to reduce a property of an individual system to a property of an ensemble, which looks more like a mathematical construct than a real physical property. The problem is exacerbated by the fact that the account entails type-identities between the reduced and the reducing quantities, which for Lewis reflect type-identities in the world. The puzzle is thus how a property of an individual system could be identical to an ensemble property.

This objection is specific to our particular case study,^25^ but this problem is arguably symptomatic of a more general potential issue for Syntactic Functional Reductionism. That is, the connection between theoretical functional reductionism and ontology is here very straightforward, but at the same time very strict. The relationship is, arguably, reference.^26^ Functional roles are logically formulated using the Ramsey sentence: *x* is that thing that plays a certain role, where playing a role is to satisfy certain predicates that connect that *x* with other kinds of theoretical terms in the network. If we adopt a scientific realist attitude, the way in which functionalism is connected with the world is very direct in the context of the present approach: the theoretical term, defined via the functional role, directly refers to the actual entity that plays the roles represented by the theory. Hence there is a straightforward one-to-one correspondence between theoretical terms and actual entities, which naturally matches a Quinean type of approach to the ontological commitments of theories, that fits neatly with an approach to theories that reformulates them via predicate logic such as the one underlying the Lewisian view.^27^ If the theory quantifies over thermodynamic entropy, then the theory is directly committed to its existence if it is true, and refers to it, and the same holds for Gibbs entropy. In our case, following the type of functional reduction established by Syntactic Functional Reductionism, they also turn out to be extensionally identical by necessity. We have shown how, within our case study, the strict correspondence between theoretical terms and actual entities, and especially the following ontological identifications, can raise puzzles. This gives us reasons to be sceptical about the strong formal-material link embedded in this framework.

In other words, the case study raises the following dilemma for Syntactic Functional Reductionism. On the one hand, one can reject scientific realism, thereby employing functional reductionism merely at the theoretical level. This is a viable option but, since most of the authors employing functional reductionism in the current literature are committed to a scientific realist attitude, it seems an unappealing choice.^28^ On the other hand, one can respond that theoretical functional reduction is a guide to ontological functional reduction, but maintain that we should not take the link as a straightforward entailment like the one pictured by Lewis. Supporting this second option would not be easy though. Indeed we would need a principled reason why the functional reduction of a term entails a functional reduction in the world only in certain situations and not in others. And, in general, we would need a novel story about the theoretical-ontological link within the account, different from the Quinean one which underlies the Lewisian picture. One could address part of the problem by adopting a pragmatic stance and claim that we should simply choose on a case-by-case basis when we should draw ontological commitments and ontological identifications.^29^ Still, once the ontological commitment is drawn, the type-identity relation follows and this can be regarded as a rather restrictive requirement. A *ceteris paribus* less restrictive account would be preferable.

To wrap up, this subsection has highlighted some limits of Syntactic Functional Reduction. These are not to be taken as critical issues hopelessly compromising the approach, but rather they bring to light the need to either (i) improve the view, moving to a form of Syntactic Functional Reductionism which is less Lewisian and e.g. employs a different formalism than first-order logic (and in this sense the present discussion can be taken as a starting point for future works in this direction), or (ii) move to a different approach. The second strategy is the focus of the next section.

## Semantic functional reductionism

Semantic Functional Reductionism constitutes an alternative to the Syntactic framework in providing a model of functional reductionism. It combines the general functional reductionist approach to inter-theoretic reduction with a model-based stance on scientific theories, and a structuralist conception of reduction as a relation between models, usually expressed mathematically. Reduction is thus characterised in terms of the functional realisation of certain (mostly mathematical) roles in the upper-level theory’s models by the theoretical elements in the lower-theory’s models. This approach is arguably a (functionally-based) improved version of the structuralist account of reduction. Especially within physics, the framework takes the mathematical formalism in which theories are expressed at face value, and uses maths and mathematical models to specify the functional roles. Because of this, the view does not run into the issues raised previously against the Syntactic approach, since it (i) does not require logical translation of the mathematical formalism in which the theory is formulated, (ii) accounts for approximation using a notion of approximation between models, and (iii) allows for a more flexible approach to ontology replacing the Quinean stance on ontological commitment with a relation characterised in terms of representation between the models and the world.

Section 4.1 introduces Semantic Functional Reductionism, building on the semantic view of theories and the related structuralist account of reduction, in particular in the form defended by Rosaler (2015, 2019b). Section 4.2 further develops the view by showing how it can account for Robertson’s case study of functional reduction and by discussing the advantages of the view.

### Introducing semantic functional reductionism

Given that Semantic Functional Reductionism crucially draws on the semantic approach to theories and the related account of structural reductionism, we start by introducing these two notions.

Let’s start with the former concept. In the context of physics, which is the focus of the essay, the semantic approach takes theories as constituted by sets of models that are mainly mathematically formulated, in the sense of ‘model’ employed by physicists.^30^ For the purpose of this paper, we shall adopt a rather minimal conception of the semantic approach.^31^ Our general take on the semantic approach is roughly represented by the combination of these two claims by Ladyman et al. (2007, p. 118): “(a) The appropriate tool for the representation of scientific theories is mathematics; (b) The relationships between successive theories, and theories at different scales whether spatio-temporal or energetic, are often limiting relations and similarities of mathematical structure (formally captured by structure-preserving maps or morphisms of various kinds), rather than logical relations between propositions”. As we shall see, the Semantic Functional Reductionist framework follows these principles by expressing functional roles via the models of the theories and functional reductive relations as structural relations between models. It should be stressed that we are not claiming that mathematical tools and modelling notions are totally unavailable to the syntactic view, but rather we wish to stress the difference between the approach to theories adopted in this section and the specific logic-based syntactic approach that underlies the Lewisian version of Syntactic Functional Reductionism presented in Sect. 3.^32^

Moving on, according to the structuralist account of reduction, reduction obtains by virtue of relations of (approximate) instantiation between theoretical structures belonging to different models.^33^ The view has been endorsed by Suppes, who claimed for instance that “the thesis that psychology may be reduced to physiology would be for many people appropriately established if one could show that, for any model of a psychological theory, it was possible to construct an isomorphic model within physiological theory” (Suppes, 1967, p. 59).^34^ The relation of isomorphism has been considered to be too strong in the subsequent literature, but the notion of reduction as a model-model mathematical relation has remained the hallmark of the approach. For example, in the passage quoted above, Ladyman et al. (2007) talk about reduction as a link between mathematical structures in terms of structure-preserving mappings or ‘morphisms’, and Wallace (2022, p. 357) argues that “reduction is [...] the realizing by some substructure of the low-level theory’s models of the structure of the higher-level theory’s models”, where “the lower-level theory instantiates the higher-level one if (roughly) there is a map from the lower-level state space to the higher-level state space that commutes with the dynamics and leaves invariant any commonly-interpreted structures (for instance, spacetime structure) in the two theories”. Notice that this view characterises reduction as a *primarily local* relation, that takes place between specific models, that is it regards specific parts of the theories. Global theory-to-theory reduction is thus derivative of local model-to-model reductions. Moreover, the relations of instantiation or morphism between the models are not exact but approximate (for the same reasons expressed earlier in Sect. 3.2), but approximation is here a relation between the models standing at different levels, and not between the higher-level theory and its own corrected version.

Having presented the semantic approach and structural reductionism at a general level, we start introducing Semantic Functional Reductionism by looking more closely at the specific model-based structuralist account of reduction proposed by Rosaler (2015, 2019b) and show how we can build on it to develop a novel form of functional reductionism alternative to Syntactic Functional Reductionism. Rosaler’s account is particularly suited to lay the grounds for a semantic approach to functional reductionism. He claims:

Theory $T_{h}$ reduces$_{T}$ to theory $T_{l}$ iff for every system *K* in the domain of $T_{h}$ – that is, for every system *K* whose behavior is accurately represented by some model $M_{h}$ of $T_{h}$ – there exists a model $M_{l}$ of $T_{l}$ also representing *K* such that $M_{h}$ reduces$_{M}$ to $M_{l}$ (Rosaler, 2015, p. 59),

where a low-level model reduces a high-level model if “the low-level model accounts for the success of the high-level model at tracking the behaviour of the system in question”. More precisely, the strategy requires that “for every physically realistic solution $x_{h}(s)$ of the high-level model $M_{h}$, there exists a solution $x_{l}(s)$ of the low-level model $M_{l}$ such that $B(x_{l}(s))$ approximates $x_{h}(s)$ to within a margin of accuracy that is at least as small as the margin within which $x_{h}(s)$ tracks the relevant features of the system *K*” (Rosaler, 2019b, p. 293), where *B* is some function mapping solutions in the low-level state space to solutions in the high-level state space.^35^

Here is an example to illustrate this approach. A semi-classical model for a point-particle system can be mathematically matched with a quantum model of the same system, under the right conditions. Thanks to the Ehrenfest theorem, we can derive Newton’s law from the Schrödinger equation for the system if the particle is highly localised in space. This means that, within the quantum model, the centre of the localised wavepacket has a trajectory in configuration space that is (to a high approximation) identical to the trajectory in configuration space of a point particle of mass *m* within classical mechanics (in the Hamiltonian formulation). Thus, the trajectory of the wavepacket can be practically considered as a solution to the classical dynamic equation for a classical particle, and we can draw a map between the quantum and the classical models defined over the respective state spaces. Notice that, as we are considering highly localised wavepackets for the purpose of reduction, the low-level state space at stake is the subset of the quantum model’s Hilbert space consisting of narrowly localised wavepackets.^36^

Taking stock of this, our aim is to show how functional reductionism, as formulated according to Semantic Functional Reductionism, can build on and improve this view. To begin with, functional reductionism can be broadly characterised in the formal mode as the view that a theory T can be reduced to another theory T* in virtue of the fact that theory T* embeds theoretical elements (e.g. quantities, systems) which can play the theoretical (formal) roles of the theoretical elements belonging to T. The thesis of Semantic Functional Reductionist is then that to establish theoretical functional reduction we need to find lower-level mathematical structures, systems, variables, or quantities playing approximately the same theoretical roles (i.e. roles in the models) of upper-level mathematical structures, systems variables or quantities in the upper-level model. This is a local and mainly mathematically couched way of expressing the idea that functional reduction proceeds by identifying the lower-level realiser for an upper-level role: in our case, the role is spelled out in mainly mathematical terms as a role in the models, and the realiser is a piece of mathematical structure. More formally, functional reduction of an upper-level theoretical element to a lower-level theoretical element takes place if we can draw a mapping from the latter to the former, where this operation maps elements that approximately play the same roles in the models of their respective theories describing the same systems. Putting this schematically, and making more precise the notion of ‘approximately playing the same role’:

1. Theoretical elements (e.g. quantity, system, structure) in the models of the top-level theory are characterised via their role within those models, as (e.g.) those quantities that approximately evolve in a certain way as expressed by the theory’s mathematical models.
2. Bottom-level theoretical elements are identified that behave approximately like the upper-level ones in the same situations, i.e. evolve approximately in the same way within the models describing the same kind of situations.
3. A mapping can be drawn between the elements: the bottom-level elements can be said to satisfy the top-level models just like the top-level elements.

This is close to Rosaler’s and Wallace’s approaches presented above but adds a functionalist aspect to the reduction process. We should also stress that one-to-one mappings between specific quantities are not a prerequisite of the approach; this is different from what happens within the form of Syntactic Functional Reduction discussed in Sect. 3. At this stage, we keep the account as minimal as possible, to make it more flexible and easier to tailor on a case-by-case basis. We take this as an upside to the account. Section 4.2 shows more rigorously how the approach can be applied.

Semantic Functional Reductionism thus shares Rosaler’s notion of reduction—as the recovery of upper-level real behaviour from the lower-level, represented in terms of model-model relations—and reformulates this in functional reductionist terms. But here is a crucial point. A reason for preferring functional reductionism here is that it gives a *justification* for Rosaler’s structuralist approach, or at least it exposes an implicit assumption: the reason why, to reduce a classical model to a quantum model, all we need to do is providing an account of how the quantum model can represent the behaviour described by the classical model, is given by the functionalist thesis that ‘being a classical system’ *just means to perform certain roles within the model of classical mechanics*. Recall the example of structural reduction presented above. We claimed that, for certain kinds of physical systems, i.e. highly localised systems, we can build mathematical mappings between the state spaces for the same system within the bottom and the upper theories’ models. That account of reduction is structuralist in the sense that we provide an asymmetrical inter-level mathematical map between the two models. However, it may be asked *why* this particular mapping ensures reduction, i.e. why the mapping provides a reason to believe that we can recover the classical system from the quantum one. Adopting a functional reductionist version of the structuralist approach to reduction provides us with the justification: the condition for being a classical system is to play a certain role in the classical models, and the mathematical mapping at stake shows exactly that the quantum system can indeed evolve like the classical one. This example highlights the intuitions behind Semantic Functional Reductionism and the sense in which the approach can be taken as an improved form of structuralist reduction. In the following, we make the account more precise by analysing our main case study and by discussing its virtues.

### Thermodynamics and semantic functional reductionism

We reconsider now the case study of Sect. 2. In Sect. 3.3 we analysed it with respect to Syntactic Functional Reductionism. We show here how the Semantic Functional Reductionist framework can accommodate the case study, to present in more detail how the approach works and its differences from Syntactic Functional Reductionism. We go through the same points discussed in Sect. 3.3 with respect to the Syntactic account. Hence we consider first how the account works at the formal level, and then discuss the theoretical-ontological link within this framework.

Let’s start by showing how the Semantic approach can be applied to our case study:

1. Take $S_{TD}$, as introduced in thermodynamics. This quantity can be characterised as that function *f* that approximately evolves in a certain way within certain processes. In adiabatic models of quasi-static processes, this is a function *f* that is constant, as per Eq. (4). In those adiabatic models representing non-quasi-static processes, the function *f* does not decrease, as per Eq. (6). Furthermore, within thermodynamic models of neighbouring equilibrium states, *f*’s evolution is determined by the evolution of energy and volume as expressed in Eq. (13).
2. Take $S_{G}$, as introduced in statistical mechanics. Similarly to Step 1, Gibbs entropy can be characterised as that function *g* approximately displaying a certain evolution in certain processes. In particular, in quasi-static processes *g* does not change, as per Eq. (11), while increases in rapid non-quasi-static processes, as shown by Eq. (12). Furthermore, if the external parameters are changed slowly enough, *g*’s evolution is determined by Eq. (14).
3. Since *g* and *f* evolve in the same way in the same kind of processes, as expressed by the models of the theories for quasi-static and non-quasi-static processes, and instantiate the same functional interdependencies as expressed within 13 and 14, a mapping $g \mapsto f$ can be drawn and functional reduction of $S_{TD}$ to $S_{G}$ is established.^37^

Let’s analyse this approach, starting with the theoretical side of the account. One sharp contrast between a Semantic Functional Reductionist version of the case study and the (Lewisian-based) Syntactic one concerns how the functional role is characterised within the account. In the latter approach, functional reduction starts with the logical translation of the higher-level theory and the formulation of the $S_{TD}$ theoretical role via Ramseyification in terms of formal logic. On the contrary, the starting point of Semantic Functional Reductionism is to get rid of those steps. The privileged tool for representing scientific theories is maths, and we should just mainly stick with maths in specifying the functional roles. Thus, as shown in the three steps above, the approach of Semantic Functional Reductionism in dealing with our case study is to maintain that the functional roles we have to identify thermodynamic entropy with are just the mathematical roles that appear in Sect. 2. In other words, thermodynamic entropy is that mathematical function that bears those mathematical relations within thermodynamic models of thermally isolated systems, in thus-and-so conditions. Therefore, any bottom-level piece of mathematical structure in the lower theory’s models that can fulfil those mathematical relations is said to functionally realise—and reduce—the thermodynamic entropy. Of course, exact fulfilment is not required: approximate realisation of the mathematical role is enough, and that very approximation is mathematically expressed. In other words, we just need the bottom-level model to approximate the upper-level one at the mathematical level.

The upshot is that there are crucial differences between the Syntactic and the Semantic frameworks considered here. First, there is no need for logical translation within the present account: as far as the mathematical presentation of the functional reduction carried out in Sect. 2 is clear enough to show how reduction works, we should just take this at face value. That is, we can account here for the case study at stake simply by reading off functional reduction from the mathematical presentation provided by Robertson. This renders reduction comparatively easier to model. Second, the Semantic framework deals with approximation and approximate reduction in a straightforward way, directly exploiting the mathematical way of representing approximation which is employed by physicists. This distinguishes this framework from the Syntactic one, and makes it preferable to the latter, as it achieves the same goal in a simpler way.^38^

Moving to the ontological aspects of the framework, we argue that the Semantic framework fares better than the Syntactic one, as it is more flexible. To recap, Sect. 3.3 elaborated on the link between theoretical reduction and ontology within Syntactic Functional Reductionism. That approach embeds a Quinean view concerning the ontological commitments of theories, which raises a potential problem when confronted with cases of functional reduction like the thermodynamic one. That account predicates direct reference between theoretical terms and entities and entails identity relations between the upper functionalised entity and the bottom-level realiser, providing an arguably too strict account of the ontological implications of functional reduction.

In contrast, Semantic Functional Reductionism allows for a more flexible approach. At the theoretical level, this framework adopts a model-based account of reduction, and frames functional reduction as a realisation relation between models: theoretical elements in the reducing theory’s models, such as mathematically formulated quantities, are taken to functionally realise certain patterns of behaviour described by the reduced theory’s models. Given its reliance on models, the view is less restrictive concerning the relationship between theory and ontology, and concerning the ontological consequences of theoretical reduction, because models are merely required to *represent* the world. Adapting Wallace’s (2022, p. 350) words to our context, we stress that while within the Syntactic framework “The relations that a good theory’s empirical statements have to the facts are those familiar from ordinary-language semantics: truth, reference, satisfaction”, within an approach like Semantic Functional Reductionism “a theory makes contact with empirical data by modelling them. [...] The theory/world relation here is *representation*, more akin to the relation between map and territory than that between word and object”.

Hence, when we claim that e.g. Gibbs entropy plays the role of thermodynamic entropy, we are not committed to the claim that there’s a specific property of a system denoted by thermodynamic entropy that is realised by a specific property denoted by Gibbs entropy and that they turn out to be ontologically identical properties. Rather, the Semantic approach just claims that the physical systems represented by the models of statistical mechanics can be modelled accordingly to the thermodynamics models under the right conditions and a mapping can be drawn between the quantities.^39^ Because of this, we are not committed to any specific view about the kind of relations between the physical systems, and thus we are not forced to endorse identity relations between them, and we are also free to be selectively realists about the type of entities our theories represent. Once again, this happens because we move from an account where the theory/world relation is reference between predicates and properties (and we argued how that feature makes Lewisian functional reductionism restrictive) to a theory/world relation relying on model-based representation. We obtain an account that is flexible enough to let us decide case by case and which does so for a principled reason, not running into a dilemma like the one we raised against Syntactic Functional Reductionism.

Before concluding, it is worth pointing out that the flexibility and the reliance on models that make the Semantic approach to functional reductionism more liberal and more straightforward, as it does not require the regimented translation into logic on which Syntactic Functional Reductionism builds, could also prompt possible drawbacks for the approach. First, the model-world relation of representation is largely left under-specified, and thus the ontological implications of inter-theoretical reduction are less clear within this approach than in the Syntactic one. Second, as admitted by Wallace (2022, p. 352) while discussing the advantages of the semantic view of theories in dealing with issues such as approximation, “it’s certainly true that we have a fuller and more carefully developed philosophical analysis of the semantic notions appealed to in the language-first [i.e. syntactic] view than we do of math-first-style [i.e. semantic-style] scientific representation”. We elaborate on this and related points in the next section.

## Gauging the two alternatives

**Table:** 

	Syntactic functional reduction	Semantic functional reduction
Theories	Syntactic	Semantic
Theory reduction	Nagelian: logical derivation	Structural: model-model
Functional roles	Logically formulated	Mainly maths formulated
Scope	Local or global	Primarily local
Ontology	Quinean approach	Representation relations

We sum up the previous sections to compare the two forms of functional reductionism introduced and review the advantages and drawbacks of each view beyond the particular discussion that we carried out around the case study of the paper.

Syntactic Functional Reductionism, which builds on the only full-fledged functional reductionist account in the literature, models the account as an improved and *sui generis* form of Nagelian reduction, cast within the syntactic approach to theories discussed above, where functional roles are logically formulated as we specified. Semantic Functional Reductionism, which is a novel introduction, provides a functionalist and upgraded version of structuralist reduction, where scientific theories are based on models and functional roles are accordingly (mostly) mathematically formulated. Both views can be classified as either local or global approaches to reduction, even though the Semantic one is markedly more locally scoped. The table above sums up these features, roughly representing the two forms of Syntactic and Semantic Functional Reductionism presented in the paper. As we shall stress in a moment, they can be taken as the endpoints of a possible *spectrum* of positions.

Discussing the case study of thermodynamics has allowed for a more detailed presentation of these frameworks and a comparison between them. Syntactic Functional Reductionism enjoys the following advantages: it provides a rigorous framework; embeds a clear link with ontology and with ontological reduction; can be easily endorsed by those that already support the syntactic view and/or Nagelian reduction. At the same time, it presents the following drawbacks: it requires a translation of theories into logic; faces issues related to approximation; the link it imposes between theoretical reduction and ontology, and the one-to-one mapping between terms in different theories, are very rigid and demanding. On the other hand, Semantic Functional Reductionism has the following merits: it is a more liberal framework with respect to the ontological implications of reduction; it does not require logical translations, as functional roles are directly extracted from the models and functional reduction is mainly mathematically formulated; makes dealing with approximations easier; can be easily endorsed by those that already support the semantic view and/or structuralist reduction. However, we also concede that the high flexibility of the account (both in terms of theoretical reduction and link with ontology) can be considered a drawback, compared with the rigour of the Syntactic framework.

Overall, the last sections bring to light the strengths and weaknesses of each approach. Rather than completely ruling out one approach or the other, the paper delved deeper into the Lewisian approach while also proposing a possible alternative, eventually providing a classification that can bring clarity to the current and future literature on functional reductionism, and most importantly providing a starting point for both a new version of Syntactic Functional Reduction and for a novel alternative approach. Or, for a hybrid version stemming from a synthesis of the two approaches.

Along these lines, a crucial point should be stressed concerning the relationship between Syntactic Functional Reductionism and Semantic Functional Reductionism as represented in the paper. That is, the distinction between the two accounts is more of a matter of degree, rather than a clear-cut distinction. Indeed, we can sketch here (i) ways in which the Lewisian basis underlying the Syntactic approach could be embedded into the Semantic approach, and (ii) a sense in which Semantic Functional Reductionism can be said to incorporate syntactic aspects. On the one side, the Lewisian approach involving functional definitions and cross-theoretical identifications could also be predicated in mainly mathematical terms along the lines of the Semantic approach. That is, instead of defining terms via a logically formulated Ramsey sentence, we could formulate the Lewisian definitions in terms of the roles in models, thus via mathematics. Another way to do that is to replace the logic employed in the Lewisian approach with a more adequate formalism e.g. based on type theory.^40^ A form of Syntactic Functional Reductionism developed along these lines could be able to appeal to a different approach regarding the theory/world relationship more akin to the one presented above within the Semantic approach, and thus possibly employ a more flexible approach to ontology. On the other side, drawing from Wallace (2022, p. 350), it would be open to an advocate of the Syntactic approach to argue that “extracting coherent content from physics requires substantial reconstruction along language-first [i.e. syntactic] lines”, and thus the Semantic approach to functional reductionism could be said to shade into Syntactic Functional Reductionism in this respect.

That being said, even if the distinction is not a clear-cut one, the classification defended in the paper is still useful and important as it carves important differences between two possible approaches to functional reductionism, and highlights the many different distinct features making up functional reductionist approaches. Rather than eliminating the distinction we thus propose to take it as a basis for future developments.

## Conclusion

Functional reductionism is a candidate account for scientific reduction, that provides an alternative to more standard approaches like Nagelian and structuralist reductionism, and that has recently been fruitfully applied to inter-theoretic reductive relations between theories like thermodynamics and statistical mechanics, classical mechanics and quantum mechanics, and general relativity and quantum gravity theories. Its potential value is demonstrated by those applications, but the view is still underdeveloped in several respects. This paper offers a thorough analysis of this approach by exploring two frameworks providing two fully developed alternative models of functional reductionism, which improve and clarify the view. The paper thus brings the functional reductionist approach to theoretical reduction to a higher level of clarity and provides a more complete picture of how this account works with respect to both theoretical and ontological reduction, contributing to making functional reductionism a viable model for reduction.

## Footnotes

1 Following Rosaler (2015, 2019b) we interpret the high-level/low-level distinction in very loose terms. Instead of high and low levels we can for instance talk about the domain of the reduced theory as opposed to the domain of the reducing theory, or we can talk about broader-scoped and narrower-scoped theories. In this sense, our discussion of reduction is able to capture also cases of inter-theoretic reduction between what are arguably same-level theories, such as general relativity and Newtonian mechanics. Nothing we say about reduction presupposes the idea that reality is ordered in a hierarchy of levels.

2 The value of functionalism in physics has been also recently defended e.g. by Knox (2019) and Wallace (2012, 2022). Functional reductionism has been discussed also by Albert (2015). Moreover, Lewis’ and Kim’s works mentioned above seek to characterise reduction across the sciences in ways that draw on functionalist intuitions.

3 We shall refer to ‘functional reductionism’ to talk about the general approach to reduction and to ‘functional reduction’ to refer to specific instances of the approach. Functional reductionism is an approach to modeling cases of reduction and remains neutral with respect to stronger views, such as the possibility of reducing everything to some fundamental level.

4 The second law of thermodynamics, according to Clausius’ statement, says that it is impossible for heat to spontaneously move from a lower-temperature reservoir to a higher-temperature reservoir.

5 We consider thermally isolated systems and reversible processes.

6 Cf. Schroeder (1999).

7 We slightly deviate here from Robertson’s presentation, for simplicity of exposition. See e.g. Tong (2012) for an introduction.

8 If we make a measurement of the energy of the system then the probability of finding ourselves in an energy eigenstate $|\phi \rangle$ is $p(\phi )=\langle \phi |\rho |\phi \rangle$, and for energy eigenstates this is indeed just (8).

9 Provided that we have adopted a new canonical ensemble tailored to the process and different from the starting one, and we have abstracted away certain details.

10 See Callender (1999) on this topic.

11 For further discussions about the advantages of functional reductionism see for instance Lam and Wüthrich (2018), Butterfield and Gomes (2022, 2023), Lorenzetti (2022).

12 Huggett and Wüthrich (2021) discuss the Lewisian approach in the context of the functional reduction of spacetime, while Lorenzetti (2022) applies this framework to Wave Function Realism, developing a functional reductionist account relating classical and quantum mechanics. See also Lorenzetti (2023) for a recent discussion of Lewisian functionalism.

13 See Kim (2005), Lewis (1970), Butterfield and Gomes (2023). See also the debate between Marras (2005) and Morris (2020) on the topic of bridge laws. See also Crowther (2018) and Palacios (2023) for recent discussions of Nagelian reduction in physics.

14 In this step the domain is the one of the upper-level theory.

15 Here theorem is used in the standard logical sense. $T[\rho _{1}...\rho _{n}]$ being a theorem of *T** is important because it means that we can derive within *T** the laws of the upper-level theory with $\rho _{1}...\rho _{n}$ as realisers. This logical derivation step is a crucial part of Nagelian reduction as we shall stress.

16 Here the domain is that of *T**, showing the two theories share at least part of their domains.

17 See Halvorson (2019), Winther (2021) for an introduction. See Lutz (2012) and Halvorson (2013) for a recent defence of the syntactic approach; and Suppes (1961, 1967), Suppe (1974), Van Fraassen (1980), Ladyman et al. (2007), Wallace (2022) for a defence of the semantic approach. See also Lutz (2017) against the distinction between syntactic and semantic and a partial defence of the former.

18 Cf. Ladyman et al. (2007). It should be stressed that the formal/material distinction does not imply that, within the domain of theoretical reduction, reduction is predicated merely on abstract or formal terms without empirical considerations. Rather, empirical considerations have consequences even at the theoretical level: this will be evident when we talk about the role of approximation and how to-be-reduced theories need to be modified in light of empirical considerations before reduction can take place. However, once those empirical considerations have been accounted for in the formulation of the theory, the reduction process can then be disjointed in a theoretical and an ontological side, where the former can be accepted even by someone who rejects scientific realism. In this sense, we can see how the formal/material distinction is more about scientific realism vs. instrumentalism rather than about a priori reduction vs. empirically-based reduction. On the topic see Rosaler (2019b) and Rueger (2006).

19 See Lewis (1970)[p. 428]. Lewis’ framework is indeed part of the so-called ‘Canberra plan’, a naturalistic philosophical framework (see Braddon-Mitchell & Nola, 2008, Chap. 1).

20 Indeed, within the Lewisian account of mental states, realiser functionalism turns out to be just a peculiar version of the identity theory about mental and physical states.

21 See also Schaffner (2012) and Dizadji-Bahmani (2021).

22 An exception is e.g. Baron (2022), who also raises a challenge related to approximation. More on the topic in Sect. 3.3. See also Lorenzetti (2022) on approximation in Lewisian functional reduction.

23 Even though of course Robertson is not committed to Syntactic Functional Reduction.

24 Furthermore, if one wants to embrace scientific realism, in this case they would need to provide a notion of ‘approximate truth’, which is a notoriously difficult task (cf. Wallace, 2022, p. 351). See also Baron (2022), who raises challenges regarding cashing out approximations in the functional reduction of spacetime, especially in the Lewisian approach.

25 For example, one could argue that when moving to the quantum domain, the canonical ensemble is represented by a density matrix and thus can represent a single quantum state.

26 On the topic see also Wallace (2022).

27 See Bricker (2016) for a standard presentation of the topic. As he broadly illustrates the Quinean approach: “if a theory contains a quantified sentence ‘$\exists$x Electron(x)’, then the bound variable ‘x’ must range over electrons in order for the theory to be true; and so the theory is ontologically committed to electrons.”. However, we stress that we are not committed to the claim that the Lewisian account is following Quine’s dictum to the letter, rather we refer to what is broadly taken as the ‘Quinean’ approach in the literature on ontological commitment. We should note that, for the sake of the argument, we are granting here that the Lewisian bound variables can range over thermodynamic and Gibbs entropy.

28 E.g. Lam and Wüthrich’s (2018, 2020) functional reductionist account is meant to deliver the functional reduction of real spacetime to non-spatiotemporal structures, while Albert (2015) and Lorenzetti (2022) support theoretical reduction between laws of quantum mechanics and classical laws to argue for the functional reduction of worldly three-dimensional entities to quantum wavefunctions.

29 On this topic, see e.g. Sklar (1967). Thanks to an anonymous reviewer for pointing this out.

30 Cf. e.g. Van Fraassen (1980).

31 As mentioned earlier, we don’t claim this description of the semantic view to be exhaustive, given the complexity of the debate, rather we just need a characterisation of the view that can distinguish it from the syntactic view in a way that is salient enough for our purpose of discussing functional reductionism. Likewise, we are not committed to a sharp and contentious distinction between logic and mathematics, but rather we are interested in distinguishing between functional roles characterised in the Lewisian way presented before and functional roles carved from the models of the theory taken at face value in their ‘textbook’ mathematical formulation. The distinction is clarified below, especially via the case study.

32 Section 5 elaborates on the possible modifications of Syntactic Functional Reductionism beyond the Lewisian model and possible intertwining between Semantic and Syntactic Functional Reductionism.

33 We are referring here to structuralism merely as an approach to reduction, following the terminology in van Riel and Van Gulick (2019, §2.4), not be confused with structuralism à la Ladyman et al. (2007). For a recent introduction to the ‘structuralistic model’ of reduction see also Palacios (2023)[§7].

34 See also Suppes (1961).

35 It should be also noted that the low-level domain considered for reduction is a restricted domain of the whole low-level model’s state space—more on this in the next paragraph. For further reference, Rosaler (2019b, §4.2.2) generalises this notion for non-dynamical systems as well. Rosaler (2019a) elaborates further on the account, especially for dynamical systems. Notice how this approach fits particularly well within the version of the semantic view defended by Van Fraassen (1980).

36 The last aspect is an additional feature that makes Rosaler’s account more refined than the structuralist approaches mentioned earlier. See Rosaler (2015, p. 63; 2018) for more details.

37 It is important to stress that this is not the same kind of mapping employed by Rosaler and Wallace in their accounts, which are mappings between state spaces. The connection between this approach and theirs is based on the fact that *g* and *f* evolve in the same way.

38 Furthermore, if we endorse scientific realism, we don’t face any problem related to the fact that the models are just approximate: since the relation between models and world is just one of representation, we don’t need to deploy a notion of *approximate truth* (see ft. 24).

39 It should be stressed that, beyond this specific example, as pointed out in the previous section, the mappings do not have to be one-to-one mappings between single specific quantities.

40 It is debatable whether this view should be regarded as ‘syntactic’ or ‘semantic’, but, since we are here stressing that the distinction can turn out to be blurry, we see this specific distinction as simply linguistic.
